# Supplementary material for: Microring resonator-assisted Fourier transform spectrometer with enhanced resolution and large bandwidth in single chip solution
Source: Nat Commun. 2019 May 28;10:2349. doi: 10.1038/s41467-019-10282-1 (PMC6538731; doi:10.1038/s41467-019-10282-1)
Supplement: Supplementary file 1 — Supplementary Information [file 41467_2019_10282_MOESM1_ESM.docx]

**SUPPLEMENTARY INFORMATION FOR**

**Microring Resonator-assisted Fourier Transform Spectrometer with Enhanced Resolution and Large Bandwidth in Single Chip Solution**

Zheng *et al*.

**Supplementary Note 1 Thermal transfer in SOI waveguides**

The designed waveguide cross section adopted in MRR and MZI arms is 450 × 220 nm^2^ and the distance between TiN heater and the waveguide top surface is 1 μm as shown in Supplementary Fig. 1.


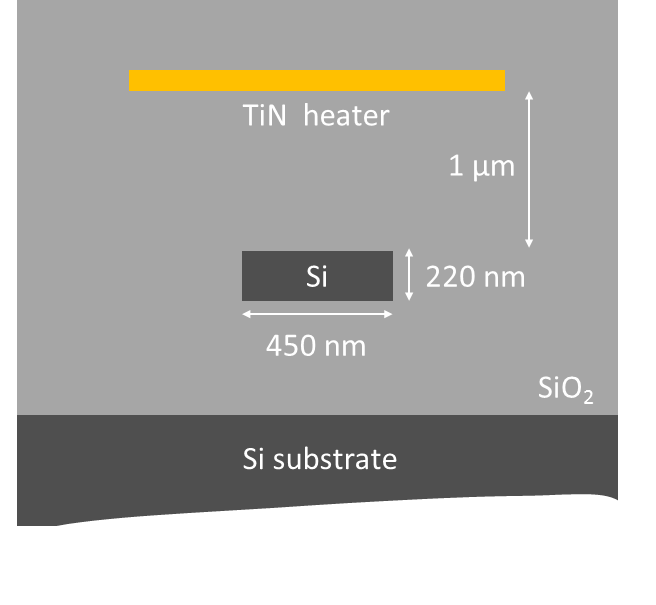


**Supplementary Figure 1.** Cross section of heater actuated SOI waveguide. TiN heater and Si waveguide are spaced with SiO_2_.

The simulation results of heat transfer in tunable MRR with and without trenches adopted using COMSOL software are shown in Supplementary Fig. 2. The static temperature *T* is linearly proportional to heating power *P* as shown in Supplementary Fig. 2a. Thus, the static temperature *T* can be written as

 (1)

where *T*_0_ is the initial temperature and the coefficient *k*_T_ is defined as the heating efficiency. Besides, the heating efficiency with trenches implemented is 1.6 times that without trenches. The temperature response is fast (10-30 μs) as shown in Supplementary Fig. 2b, while the response without trenches is slightly faster than that with trenches.


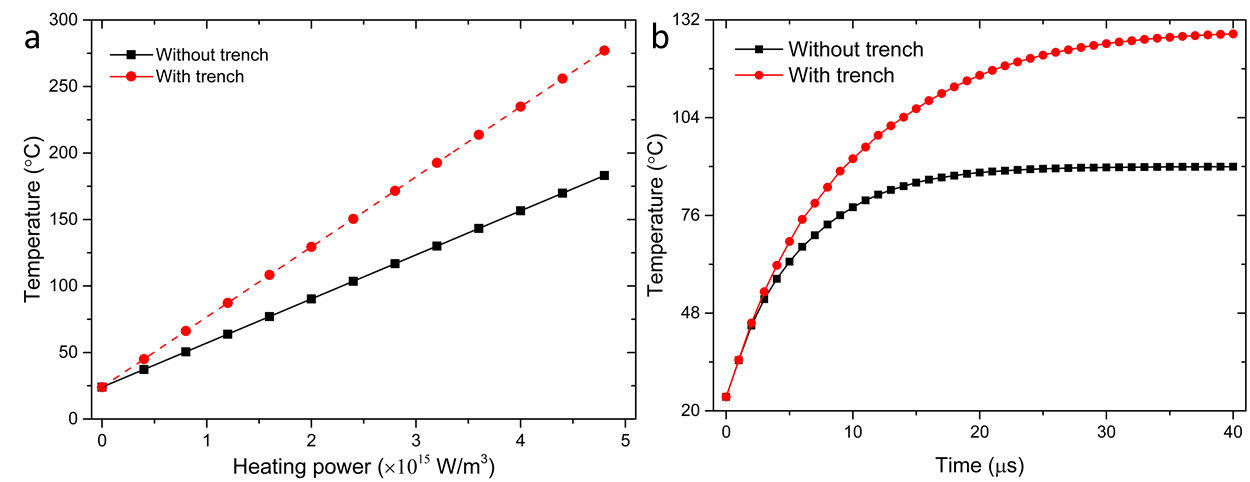


**Supplementary Figure 2.** Heat transfer in a thermally tunable MRR. **a**. Relation between the static temperature and the heating power with and without trenches. **b**. Dynamic temperature with and without trenches.

The influence of heater width on the heat transfer is also analyzed. With the same *P* applied, higher static temperature is obtained with larger heater width as shown in Supplementary Fig. 3a, which means the heating efficiency is higher with smaller heater width. There is no obvious change in temperature response time for different heater widths as shown in Supplementary Fig. 3b.


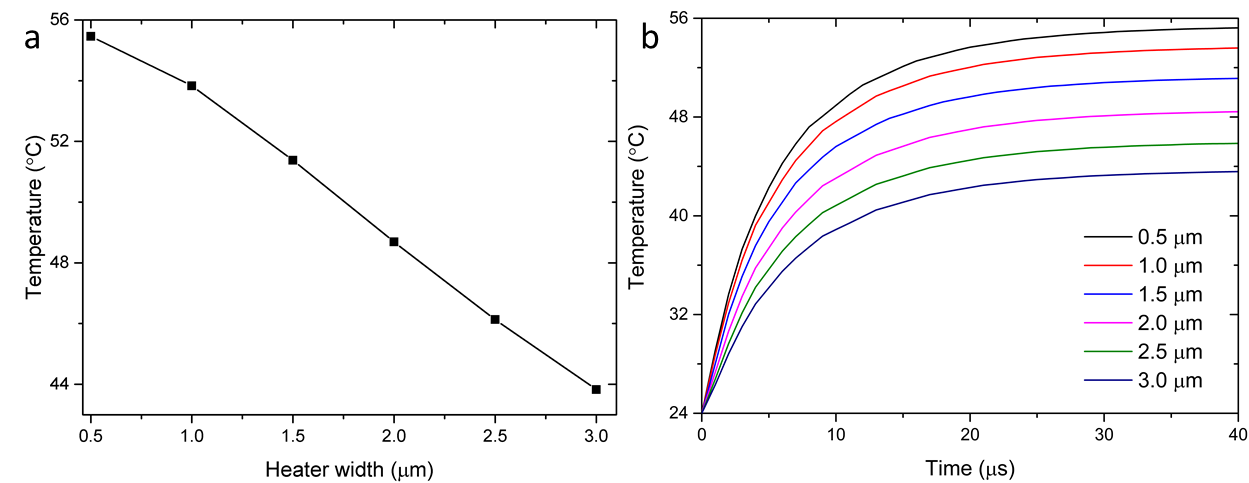


**Supplementary Figure 3.** Influence of heater width. **a**. Static temperature. **b**. Temperature response.

The electric response of TiN heater is experimentally tested as shown in Supplementary Fig. 4. The data points are well fitted with a polynomial curve (with polynomial order 2 and 99.977% fitting R-Square). Thus, by applying voltage signal to heaters, the electric power dissipated in MRR or MZI can be extracted.





**Supplementary Figure 4.** Electric response of TiN heater. The electric power is well fitted to the applied voltage on heater with a nonlinear polynomial curve. The error bars denote S.D.

**Supplementary Note 2 Thermal tuning of MRR and MZI**

The refractive index of both Si and SiO_2_ is wavelength and temperature dependent [^1^](#_ENREF_1)^,^ [^2^](#_ENREF_2). By referring to[^3^](#_ENREF_3), the calculated parameter values of waveguide dispersion, thermo-optic effect and thermal expansion around *T*_0_ = 300 K and *v*_0_ = 193.414 THz are shown in Supplementary Table 1. Sellmeier model considering both dispersion and temperature dependent thermo-optic coefficient (TOC) from[^2^](#_ENREF_2) is adopted for SiO_2_ considering the nearly constant TOC of SiO_2_. The TOC model of Si is adopted from[^4^](#_ENREF_4), while the dispersion of Si is adopted from[^2^](#_ENREF_2). The TOC of Si has a second-order dependent on temperature as shown in Supplementary Fig. 5a. The second order is neglected for temperature excursion of 100 K due to the maximum contribution of 1.2% compared to the first order contribution of 13.5% (Supplementary Fig. 5b). The thermal expansion coefficient also presents an obvious dependence on temperature[^5^](#_ENREF_5) as shown in Supplementary Fig. 6a. The third order term has a neglectable contribution (maximum 1.7%) for temperature excursion of 100 K compared to lower orders, i.e., 34.2% for the first order and 9.9% for the second order (Supplementary Fig. 6b). The effective index around *v*_0_ = 193.414 THz considering up to the third order is expressed as

$n_{eff}(v)=n_{eff}|_{v_{0}}+\partial_{v}n\Delta v+\frac{1}{2}\partial_{v^{2}}n\Delta v^{2}+\frac{1}{6}\partial_{v^{3}}n\Delta v^{3}$ (2)

Thus, the modified effective index change ∆*n*_eff_ is written as

$\begin{matrix} \Delta n_{eff}(v,\Delta T)=(\partial_{T}n+\partial_{v,T}n\Delta v+\frac{1}{2}\partial_{v^{2},T}n\Delta v^{2}+\frac{1}{6}\partial_{v^{3},T}n\Delta v^{3})\Delta T \\ +\frac{1}{2}(\partial_{T^{2}}n+\partial_{v,T^{2}}n\Delta v+\frac{1}{2}\partial_{v^{2},T^{2}}n\Delta v^{2}+\frac{1}{6}\partial_{v^{3},T^{3}}n\Delta v^{3})\Delta T^{2} \end{matrix}$ (3)

The modified arm length is expressed as

$\Delta L(\Delta T)=L(\alpha_{1}\Delta T+\alpha_{2}\Delta T^{2}+\alpha_{3}\Delta T^{3})$ (4)

The effective index difference induced by fabrication *δn* is expressed as

$\delta n(v)=\delta(n_{eff}|v_{0})+\delta(\partial_{v}n)\Delta v+\frac{1}{2}\delta(\partial_{v^{2}}n)\Delta v^{2}+\frac{1}{6}\delta(\partial_{v^{3}}n)\Delta v^{3}$ (5)

The resonance wavelength change of MRR[^6^](#_ENREF_6) is *∆λ*_r_ = *λ*_r0_*∆n*_eff_*/n*_g_, where *λ*_r0_ is the initial resonance wavelength and *n*_g_ is the group index. The subscript *r* means resonance. The resonance wavelength is thus expressed as

$\lambda_{r}=\lambda_{r0}+\frac{\lambda_{r0}}{n_{g}}\cdot\Delta n_{eff}$ (6)

Thus, the resonance wavelength is proportional to the effective index change.

| Parameter | Value | Unit | Parameter | Value | Unit |
| --- | --- | --- | --- | --- | --- |
| $n_{eff}\vert_{v_{0}}$ | 2.23 | - | $\partial_{T^{2}}n$ | 2.5×10^-7^ | K^-2^ |
| $\partial_{v}n$ | 1.1×10^-2^ | THz^-1^ | $\partial_{v,T^{2}}n$ | -4.6×10^-9^ | K^-2^ THz^-1^ |
| $\partial_{v^{2}}n$ | 4.8×10^-6^ | THz^-2^ | $\partial_{v^{2},T^{2}}n$ | 1.7×10^-9^ | K^-2^ THz^-2^ |
| $\partial_{v^{3}}n$ | -2.3×10^-6^ | THz^-3^ | $\partial_{v^{3},T^{2}}n$ | 7.0×10^-10^ | K^-2^ THz^-3^ |
| Parameter | Value | Unit | Parameter | Value | Unit |
| $\partial_{T}n$ | 1.9×10^-4^ | K^-1^ | *α*_1_ | 2.5×10^-6^ | K^-1^ |
| $\partial_{v,T}n$ | 3.5×10^-7^ | K^-1^ THz^-1^ | *α*_2_ | 8.5×10^-9^ | K^-1^ |
| $\partial_{v^{2},T}n$ | -6.4×10^-8^ | K^-1^ THz^-2^ | *α*_3_ | -2.3×10^-11^ | K^-1^ |
| $\partial_{v^{3},T}n$ | -2.0×10^-8^ | K^-1^ THz^-3^ |  |  |  |

**Supplementary Table 1.** Parameter values of waveguide dispersion, thermo-optic effect and thermal expansion. The dispersion and thermo-optic coefficients are obtained for the quasi-TE mode of waveguide shown in Supplementary Fig. 1. The partial derivative is used as$\partial n_{eff}/\partial x=\partial{}_{x}n$.


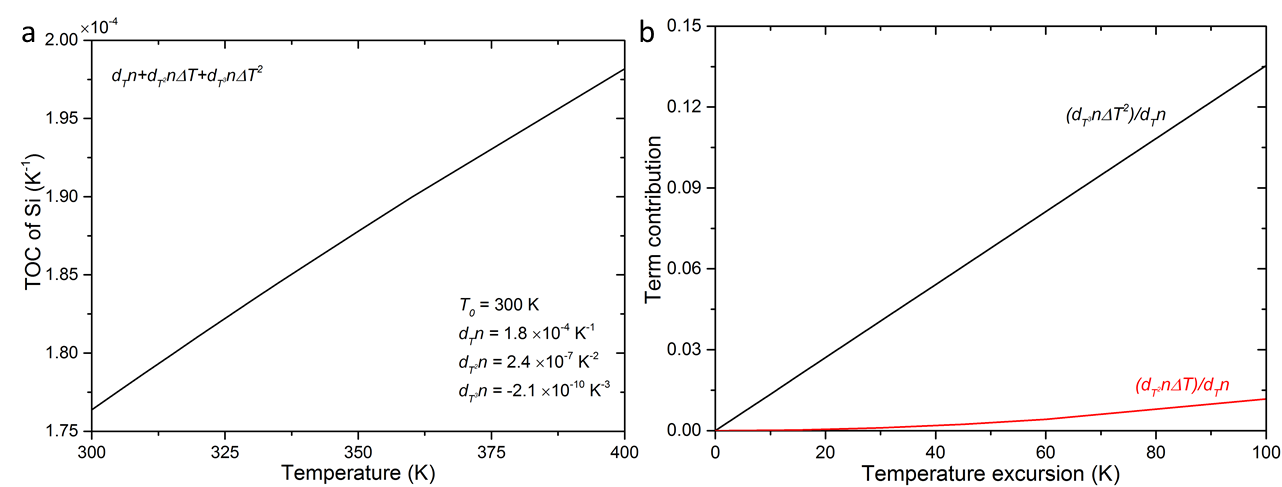


**Supplementary Figure 5.** Thermo-optic coefficient (TOC) of Si. **a**. TOC of Si has a second-order relation with temperature. **b**. Relation between term contribution and temperature excursion.


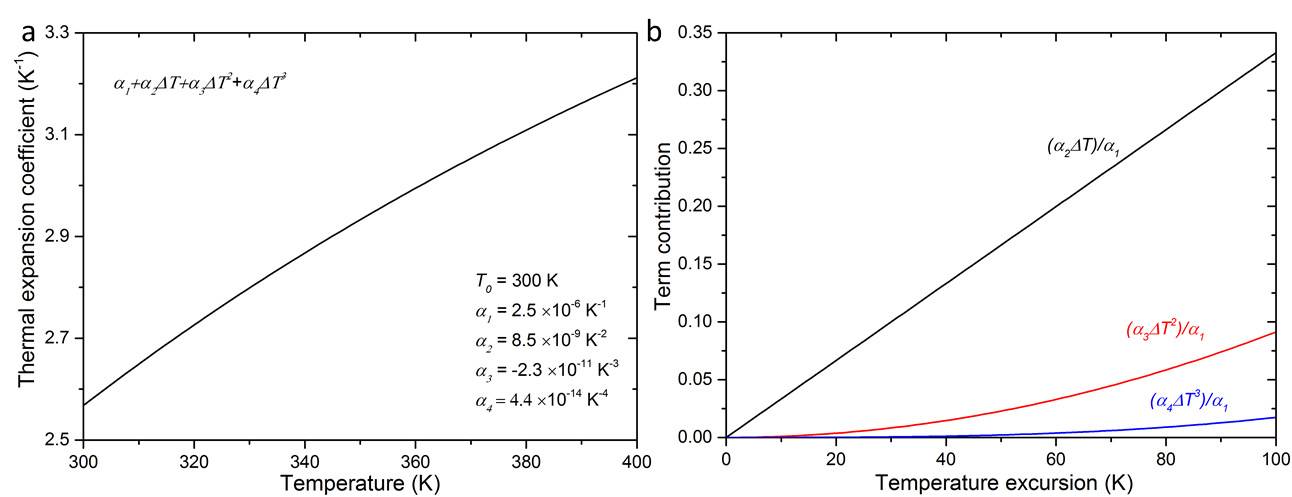


**Supplementary Figure 6.** Thermal expansion coefficient of Si. **a** Relation between thermal-expansion coefficient and temperature. **b** Relation between term contribution and temperature excursion.

The schematic of RAFT spectrometer working principle is shown in Supplementary Fig. 7. The input spectrum *I*_i_(*v*) transmits to MRR. The transmitted spectrum *I*_m_(*v*) output from drop port can be written as

$I_{m}(v)=T(v)I_{i}(v)$ (7)

where *T*(*v*) is the transmission factor of MRR[^6^](#_ENREF_6). For an input spectrum with single wavelength *I*_m_(*v*_0_) in the input port of the tunable MZI, the output intensity of MZI is

$I_{o}(\delta)=0.5I_{m}(v_{0})(1+\cos(2\pi v_{0}\delta/c))$ (8)

where *δ = ∆n*_eff_*L* is the optical path difference (OPD) of the MZI with an arm length of *L*. In practice, there are some factors that will influence the signal intensity detected by the detector. The beam splitter is not perfect to give 50% reflection and 50% transmission. Light will experience losses in beam splitters and in waveguides of MZI arms, etc. As a result, the output intensity should be modified as

$I_{o}(\delta)=0.5T(v_{0})H(v_{0})G(v_{0})I_{i}(v_{0})(1+\cos(2\pi v_{0}\delta/c))$ (9)

where *H*(*v_0_*) and *G*(*v_0_*) are wavelength dependent correction factors for imperfect beam splitters and optical losses, respectively. The coefficient 0.5*T*(*v*_0_)*H*(*v*_0_)*G*(*v*_0_) can be set to *B*(*v*_0_). Thus, the output intensity is *B*(*v*_0_)*I*_i_(*v*_0_)(1+cos(2*πv_0_δ/c*)), where the modulated portion *B*(*v*_0_)*I*_i_(*v*_0_)cos(2*πv_0_δ/c*) constitutes the interferogram. Thus, taking the modulated portion only, the output intensity for a broadband input spectrum is

$I_{o}(\tau)=\int_{-\infty}^{+\infty} B(v)I_{i}(v)\cos(2\pi v\tau)dv$ (10)

where *τ* = *δ*/*c*. After applying Fourier transform to the above equation, the input spectrum *I*_i_(*σ*) can be obtained as

$I_{i}(v)=\frac{2}{B(v)}\int_{0}^{+\infty} I_{o}(\tau)\cos(2\pi v\tau)d\tau$ (11)

Taking account of waveguide dispersion, temperature dependent TOC and thermal expansion, the phase difference between two arms is simplified to[^3^](#_ENREF_3)

 (12)

with the modified time delay

$\Gamma\equiv\tau+\gamma_{2}\tau^{2}$ (13)

and modified optical frequency

$u\equiv v_{0}+\Delta v(1+\xi_{1})$ (14)

Real time delay *τ* is linearized as modified time delay г to match the phase difference. The modified optical frequency stretches the original frequency around *v*_0_ by a factor 1+*ξ*_1_. According to the definitions of *γ*_2_ and *ξ*_1_ in[^3^](#_ENREF_3), *γ*_2_ is expressed as

$\gamma_{2}\equiv\frac{c}{L}\frac{n_{eff}|_{v_{0}}\alpha_{2}+\partial_{T}n\alpha_{1}+\frac{1}{2}\partial_{T^{2}}n}{(n_{eff}|_{v_{0}}\alpha_{1}+\partial_{T}n)^{2}}$ (15)

and the parameter *ξ*_1_ is expressed as

$\xi_{1}\equiv\frac{v_{0}(\partial_{v}n\alpha_{1}+\partial_{v,T}n)}{n_{eff}|_{v_{0}}\alpha_{1}+\partial_{T}n}$ (16)

Hence, TOC nonlinearity and thermal expansion broaden and shift the spectrum to higher frequencies. Waveguide dispersion contributes to stretched retrieved spectrum around *v*_0_^[3](#_ENREF_3)^. Replace *v* with *u* in Supplementary Eq. 10, we obtain

 (17)

Hence, the modified input spectrum is expressed as

$I_{i}(u)=\frac{2(1+\xi_{1})}{B(u)}\int_{0}^{+\infty} I_{o}(\Gamma)\cos(2\pi u\Gamma)d\Gamma$ (18)

Finally, the original input spectrum is retrieved by transforming *u* to *v*,

 (19)

The calibration of absolute optical frequency *v*, γ_2_ and ξ_1_ can be performed by a tunable laser source at different laser frequencies (on resonance and at least three)[^3^](#_ENREF_3). The time delay can be written as *τ* = *κ_τ_P*. The interferogram with a laser source input is

 (20)

where *K*(*v*) = *κ_τ_*(1 + *ξ*_1_)∆*v* + *κ_τ_v_0_* and *γ*_w_ = *κ_τ_* *γ*_2_. The coefficient *K*(*v*) and *γ*_w_ can be obtained by curve-fitting the interferograms with Supplementary Eq. 20, a nonlinear cosine function. As sown in Supplementary Fig. 8a, the mean power intensity of the interferogram is subtracted to obtain the black curve. The envelope (red) is obtained by Hilbert transform. Hence, the normalized power intensity is obtained shown in Supplementary Fig. 8b (data points in red). The curve in black is the fitting result. Hence, *K*(*v*) and *γ*_w_ are obtained by linear fitting of at least three data points as shown in Supplementary Fig. 8c and d, respectively. The parameter *κ_τ_* relating heating power to time delay is 0.245 ps W^-1^. The stretching parameter *ξ*_1_ is 0.55. The parameter *γ*_2_ is 8 ×10^-3^ ps^-1^. The parameter *η_1_* equals to (*n*_eff_|*_v_*_0_ *α*_1_+ *∂_T_n*)*L*/*c* ≈ 1.56 × 10^-14^. Hence, the maximum temperature excursion is *∆T* = *κ_τ_*·*P*_m-max_*/η_1_* *≈* 24.9 K and the heating efficiency *k*_T_ = *∆T*/*P*_m-max_ ≈ 13.8 K W^-1^. For temperature excursion of MZI less than 30 K in our experiment, the first order contribution of TOC is less than 4%.

*B*(*u*) can be obtained through experimental power calibration for each optical frequency. The same retrieval process applies to all MRR thermal tuning states. By scanning the MRR resonance wavelength shift from 0 to FSR, the original input spectrum will be retrieved by combining all retrieved sparse spectra.


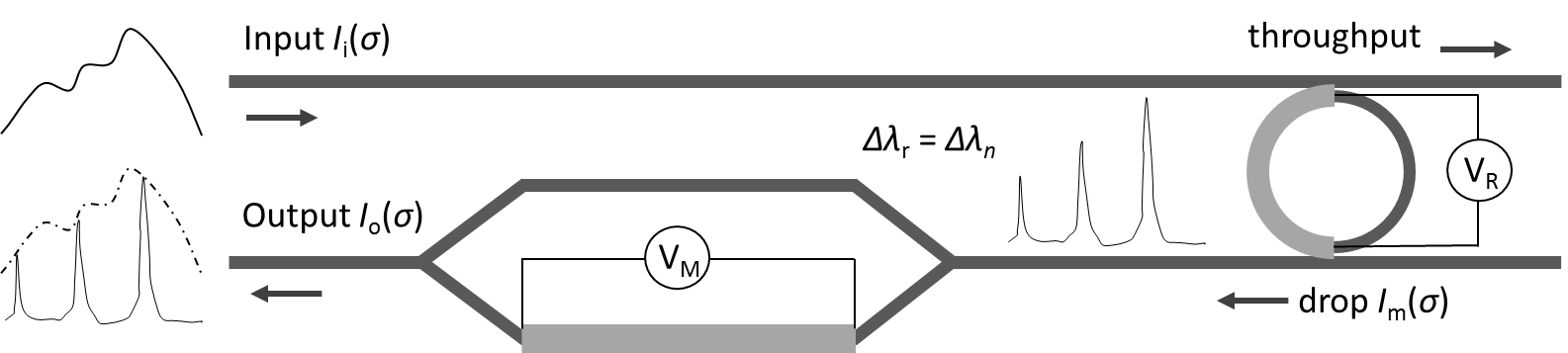


**Supplementary Figure 7.** Schematic of the RAFT spectrometer working principle. The resonance wavelength shift *∆λ* = *∆λ_n_* (0 ≤ *∆λ_n_* < FSR), where FSR is the free spectral range of the MRR.


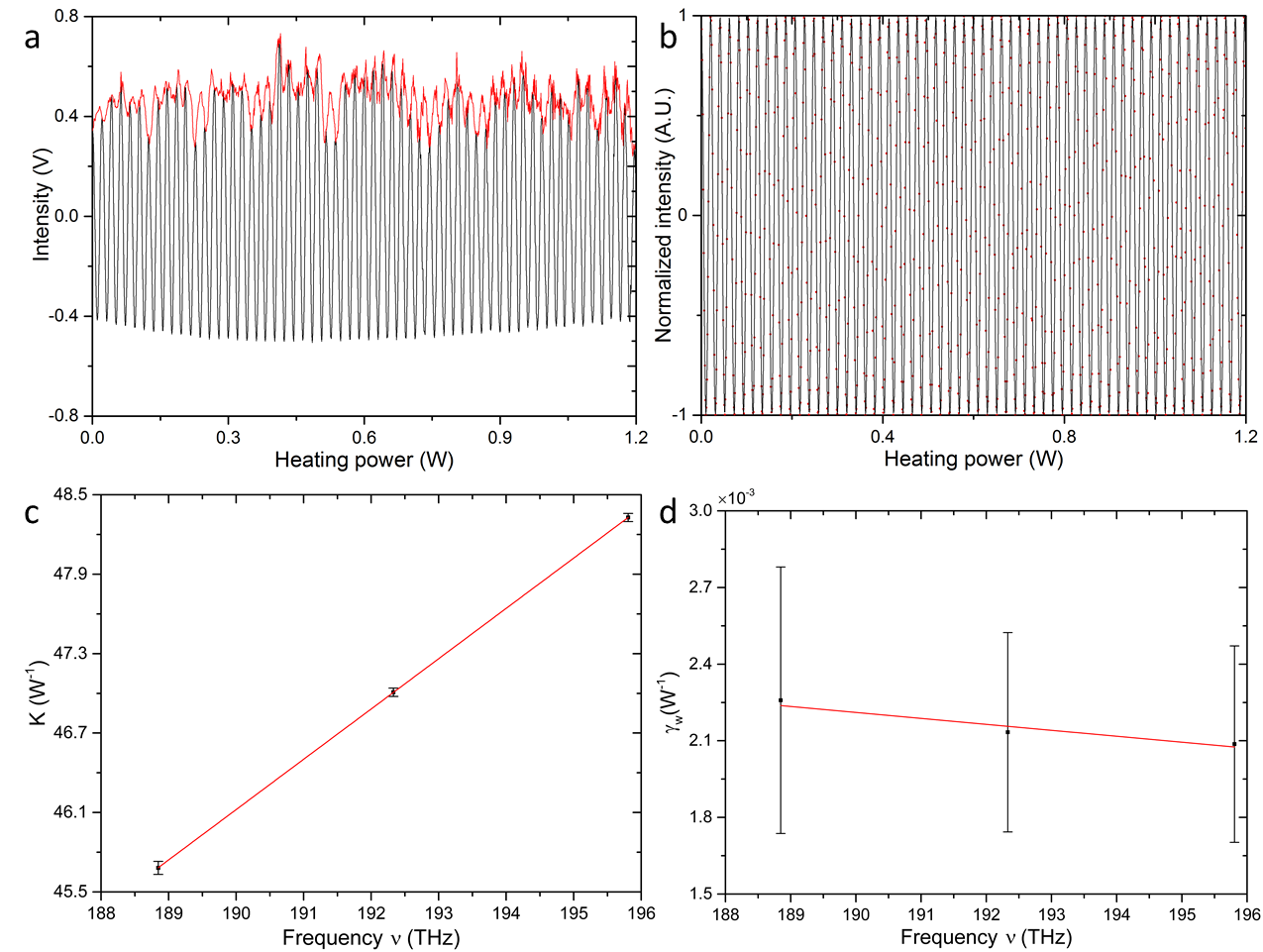


**Supplementary Figure 8.** Calibration with a tunable laser source. **a**. Interferogram (black) after the mean value is subtracted. The envelop (red) obtained by Hilbert transform. **b**. Interferogram (red) after normalization. The data are fitted with Supplementary Eq. 20. **c**. Parameter *K*(*v*) from the fitting. The error bars denote S.D. **d**. Parameter *γ*_w_ from the fitting. The error bars denote S.D.

The calibration process for broadband source measurement is as follows. A broadband ASE light source (Amonics ALS-CL) covering C and L band is used as the input. Here in our experiment, the bandwidth is 90 nm and FSR of the MRR is ~28 nm. Hence the number of the retrieved resolution elements is 3 for each tuning state of the MRR. After completing the MRR thermal tuning to cover one FSR, all the sampled interferograms from input source are obtained. There are two steps to perform wavelength/frequency calibration. The first step is to coarsely determine the frequencies of the sparse spectra by performing fast Fourier transform (FFT) to the sampled interferograms. The second step is to finely determine the frequencies according to the tuning state of the MRR. Subsequently, we normalize the retrieved power to the input power for each wavelength to obtain a normalization coefficient matrix *A* (Supplementary Fig. 9) including MRR tuning states, wavelengths and their corresponding transmission coefficients.


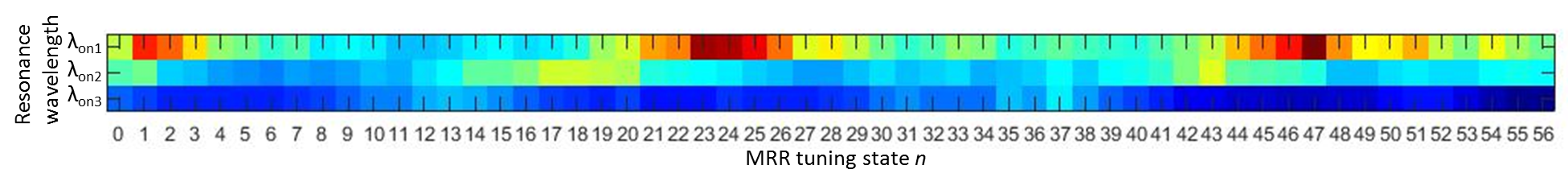


**Supplementary** **Figure 9.** Normalization coefficient matrix A for broadband spectrum measurement. It includes MRR tuning states, wavelengths and their corresponding transmission coefficients.

The simulated relation between OPD and the resolution of the tunable MZI-based Fourier-transform spectrometer *R* and heating power *P* on heater 2 is shown in Supplementary Fig. 10. Here we mainly focus on the variation trends of OPD and resolution with changing *k*_T_ and *L* and considering that the first-order term of TOC contributes far less than the zero-order term, a constant TOC is assumed in the simulation for simplicity. And the temperature excursion is fixed at a moderate value of 24.9 K. The conditions at different parameters are shown in Supplementary Table 2.


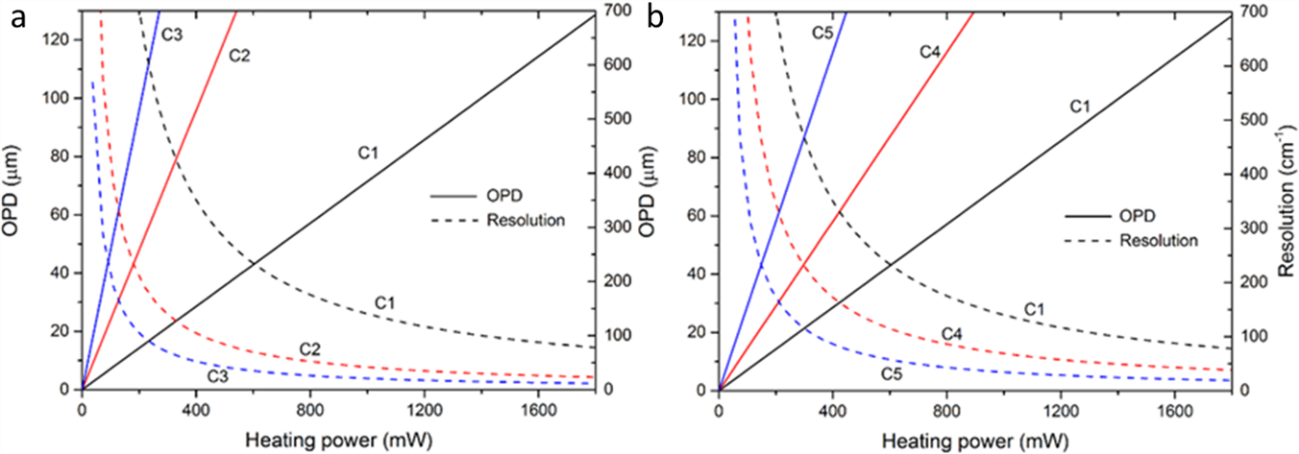


**Supplementary Figure 10.** Simulated relations between OPD, *R*, and *P*. **a** Arm length is fixed. **b** Heating efficiency is fixed.

| Conditions | Effective TOC  (K^-1^) | Heating efficiency  *k_T_* (K W^-1^) | Arm length  *L* (cm) |
| --- | --- | --- | --- |
| C1 | 1.95×10^-4^ | 14.9 | 2.46 |
| C2 | 1.95×10^-4^ | 50 | 2.46 |
| C3 | 1.95×10^-4^ | 100 | 2.46 |
| C4 | 1.95×10^-4^ | 14.9 | 5 |
| C5 | 1.95×10^-4^ | 14.9 | 10 |

**Supplementary Table 2.** Parameter values at different simulation conditions. Arm length is fixed for C1, C2 and C3. Heating efficiency is fixed for C1, C4 and C5.

Therefore, the resolution of the tunable MZI can be improved either by increasing the arm length and/or increasing the heating efficiency. The maximum arm length depends on the tolerable optical loss. For waveguide with Si core and SiO_2_ cladding adopted here, the loss rate is 1~2 dB cm^-1^, resulting in 10-20 dB loss with 10 cm length. The loss experienced in the device will add to the total insertion loss, leading to low throughput namely low SNR. One efficient way to improve heating efficiency is to fabricate isolation trenches near MZI arms to remove Si substrate to reduce heat dissipation[^7^](#_ENREF_7). Another way is to change heater material and put it on the waveguide rib[^7^](#_ENREF_7). The two approaches to improving heating efficiency are also viable for reducing heat consumption of MRR. Without the MRR, to achieve 0.47 nm resolution, the OPL should be ~102 cm at *k*_T_ = 14.9 K W^-1^ without increasing the maximum temperature excursion, which will induce >100 dB loss.

**Supplementary Note 3 Thermal isolation and compensation**

In the RAFT spectrometer, there are two parts contributing to the power consumption, namely the tunable MRR and the tunable MZI. If we assume *∆t* as the one-time scan duration and the number of scans *N* = FSR/*δλ*, the extra energy consumed to tune the MRR from one state *∆λ_n_* to the next state *∆λ_n_*_+1_ is

$P=\frac{P_{\text{r}-\max}}{N-1}\Delta t$ (21)

Hence, the total energy consumed by MRR for *N* scans can be estimated as

$P_{\text{r}-total}=0.5NP_{\text{r}-\max}\Delta t$ (22)

where *P*_r_*_-_*_max_ is the maximum heating power and equals to 35 mW in our experiment. Similarly, the total power consumed by MZI for *N* scans is estimated as

$P_{\text{m}-total}=\frac{1}{3}NP_{\text{m}-\max}\Delta t$ (23)

where *P*_m_*_-_*_max_ is the maximum heating power for MZI and is estimated to be ~1.8 W in our experiment. The one-time scan duration *∆t* is 2 s and *N* = 56 in the experiment. Hence, the total energy consumed by MRR *P*_r-total_ = 1.96 J. And the total energy consumed by MZI is *P*_m-total_ = 67.2 J. Considering the power consumption of MZI, we can employ isolation trenches near MZI arms in the chip fabrication to improve the heating efficiency. MZI testing structures are fabricated to test the influence of isolation trenches on the heating efficiency. The schematic is shown in Supplementary Fig. 11a and the SEM image of the cross section of the thermal isolation trench is shown in Supplementary Fig. 11b. The power consumption is decreased with decreasing gap between waveguide and trench as shown in Supplementary Fig. 11c. It is also decreased from 2 to 1.4 mW π^-1^ when the trench segment length *L* is increased from 40 to 53 μm. Hence, heating efficiency can be improved to maximum 12 times by reducing the gap and/or increasing the trench segment length *L*. Hence, the maximum heating power can be reduced to 1.8/12 W = 150 mW and the total energy consumed by MZI can be reduced to *P*_m-total_/12 = 5.6 J. Note that the Si substrate under the waveguide is not totally removed (Supplementary Fig. 11b), the heating efficiency can be further reduced (~8.75 times) when the waveguides are fully suspended[^8^](#_ENREF_8).


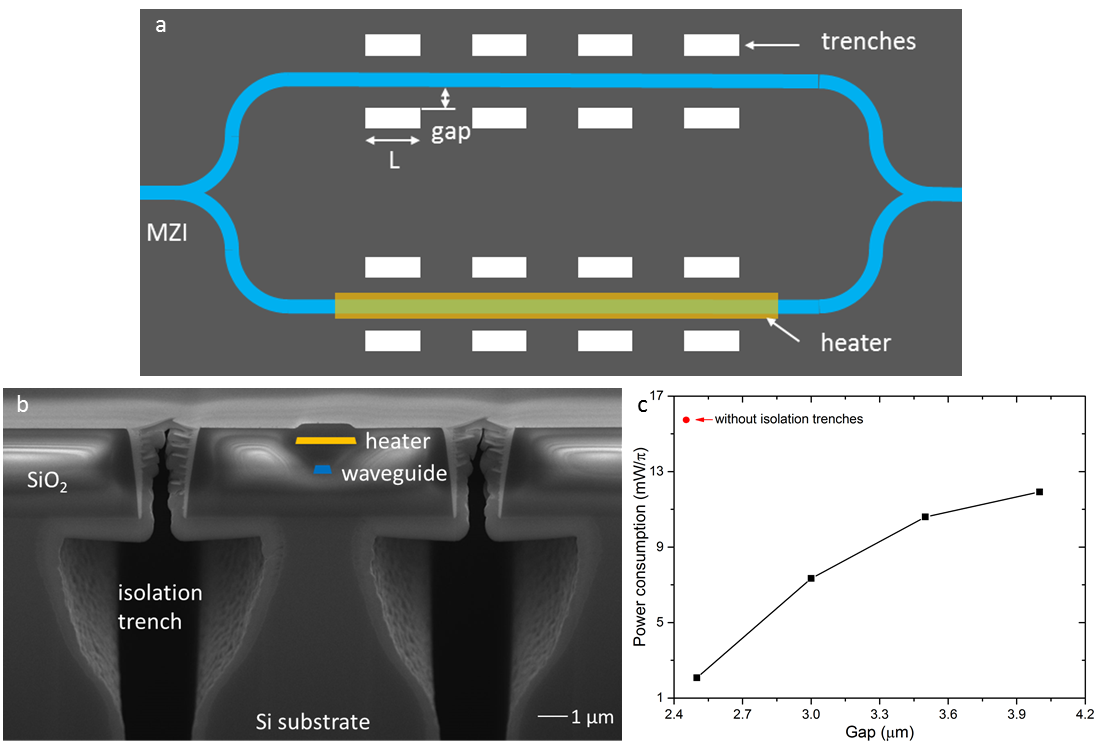


**Supplementary Figure 11.** Heating efficiency improvement with thermal isolation trenches. **a** Schematic of MZI testing structure with isolation trenches. **b** SEM image of the cross section of the thermal isolation trench. **c** Power consumption vs gap between trench and waveguide.

The thermal crosstalk from MZI heater 2 will affect the resonance position of the MRR as shown in the Supplementary Fig. 12, while there is no obvious influence on the MZI by MRR heater 1.





**Supplementary Figure 12.** Resonance wavelength shift due to thermal crosstalk. The resonance wavelength is well fitted to the heating power on heater 2. The maximum shift due to thermal crosstalk from the MZI heater is ~1 nm.

The position can be stabilized by thermal compensation. The resonance wavelength *λ*_r_ is proportional to heating power applied on heater 1, *P*_r_ (Fig. 4c), the resonance wavelength shift *dλ*_r_ can be written as

$d\lambda_{r}=B_{1}P_{r}$ (24)

where *B*_1_ is a constant. The resonance wavelength is also proportional to the heating power on heater 2, *P*_m_. Hence, the resonance wavelength shift due to MZI thermal tuning is expressed as

$d\lambda_{r}=B_{2}P_{m}$ (25)

where *B*_2_ is a constant. The parameter *P*_r_*^’^* is defined as

$P_{r}^{'}=\frac{B_{2}}{B_{1}}P_{m}$ (26)

Hence, *P*_r_*’* is the corresponding value applied on heater 1, which causes equal resonance wavelength shift by *P*_m_. Suppose the maximum resonance wavelength shift due to MZI thermal tuning when no heating power is applied on heater 1 is *dλ*_m_, the corresponding value on heater 1 to cause the same shift is written as

$P_{0}=\frac{B_{2}}{B_{1}}P_{m-\max}$ (27)

For an MRR tuning state *∆λ_n_* when resonance wavelength shift is tuned by heater 1 to *∆λ_n_*, the value of *P*_r_ on heater 1 should be

$P=\frac{\Delta\lambda_{n}}{B_{1}}+P_{0}$ (28)

Due to influence of *P*_m_, to make the tuning status stable at *∆λ_n_*, the final value of *P*_r_ after compensation should be *P* - *P*_r_*^’^*, i.e.

$P_{r}=\frac{\Delta\lambda_{n}}{B_{1}}+\frac{B_{2}}{B_{1}}(P_{m-\max}-P_{m})$ (29)

As a result, for a given *P*_m_, the resonance position can be stabilized by adjusting *P*_r_. Due to the thermal compensation, the contrast of the interferogram is drastically improved as shown in Supplementary Fig. 13.


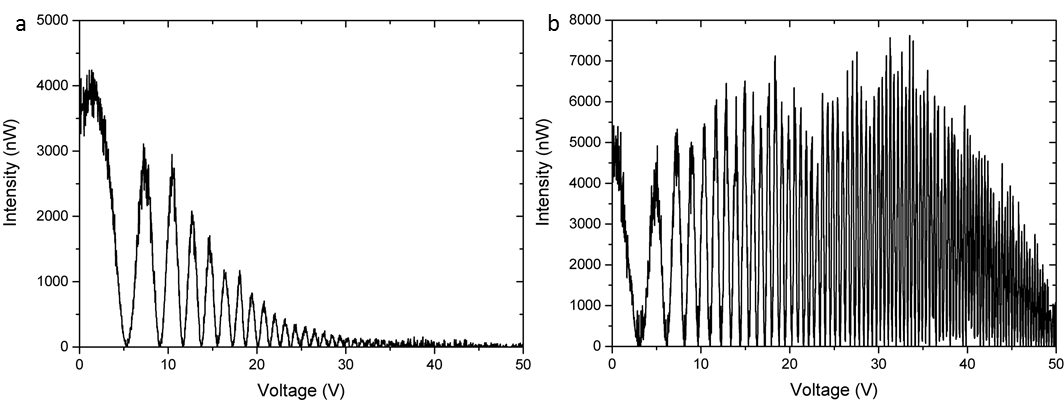


**Supplementary Figure 13.** Thermal compensation. Experimental measured interferograms with a tunable laser source input **a** before and **b** after thermal compensation.

**Supplementary Note 4 Time-multiplexing analysis**

The RAFT spectrometer factors the input spectrum to *N* (equal to FSR/*δλ*) parts, which is realized with the MRR thermal tuning. Hence, the MRR before the MZI will compromise the Fellgett advantage of a typical FT spectrometer. For BW = 90 nm, and *δλ* = 0.47 nm, the multiplex gain should be

$\sqrt{M}=\sqrt{BW/\delta\lambda}\approx13.838$ (30)

According to the definition, the multiplex gain with an MRR can be written as

$\sqrt{M^{'}}=\sqrt{m}$ (31)

where *m* is the number of FSR utilized and *m* ≈ BW/FSR. Hence, the loss in multiplex gain is

$\beta=1-\sqrt{\frac{M^{'}}{M}}\approx1-\sqrt{\frac{\delta\lambda}{FSR}}$ (32)

The relations between loss in multiplex gain *β*, resolution of the tunable MZI *R* and the number of FSR *m* are shown Supplementary Fig. 14.

The time-scale measurement is performed. For MZI thermal response, the rise and fall time are 37 μs and 60 μs, respectively, as shown in Supplementary Fig. 15a. For MRR thermal response, the rise and fall time are 20 μs and 80 μs, respectively, as shown in Supplementary Fig. 15b.





**Supplementary Figure 14.**  Relations between loss β, R and m. The loss can be reduced by choosing larger m while keeping a practical value of R.


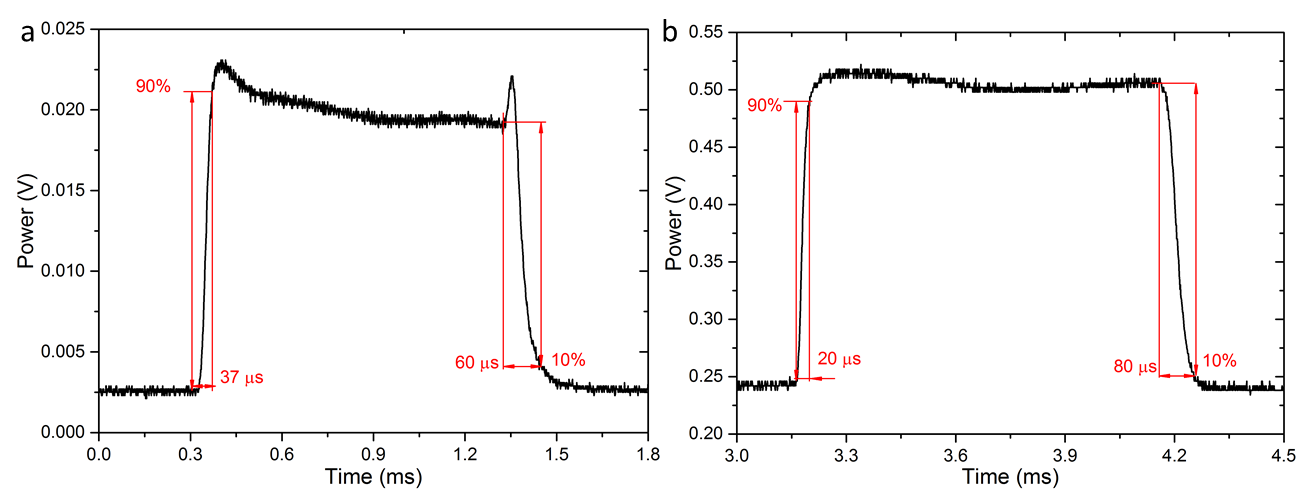


**Supplementary Figure 15.** Time-scale measurement. Thermal response time of **a** tunable MZI and **b** tunable MRR.

**Supplementary Note 5 Ring Coupler Analysis**

Higher quality factor (*Q*) value enables higher spectrometer resolution. The *Q* value for a symmetric add-drop ring is expressed as[^9^](#_ENREF_9)

$Q=\frac{\pi n_{g}L\sqrt{r^{2}a}}{\lambda_{r}(1-r^{2}a)}$ (33)

where *n*_g_ is the group index, *L* is the round-trip length of the MRR, *r* is the self-coupling coefficient, and *a* is the single-pass amplitude transmission. The *Q* value is proportional to *r* as shown in Supplementary Fig. 16a. The transmission to the drop port is expressed as[^9^](#_ENREF_9)

 (34)

where *ϕ* = 2*πn*_eff_*L*/*λ* is the single-pass phase shift. The simulated relation between the transmitted power carried by the filtered sparse spectrum (number of FSR *m* = 3) and spectrometer resolution *δλ* with a uniform broadband source input is shown in Supplementary Fig. 16b. In the experiment, the total loss of the input light is 13.92 dB, including on-chip loss of 4.92 dB and off-chip loss of 9 dB. This loss is considered in the simulation. The simulated relation between signal-to-noise ratio (SNR) and resolution at different input power values is shown in Supplementary Fig. 16c. We see that the minimum resolution value is limited by SNR requirement. In our experiment, the signal can be differentiated from noise and successfully retrieved when SNR ≥ 3 dB, which will define the minimum resolution value for each input power values as shown in Supplementary Fig. 16c. The parameter values in the simulation are shown in Supplementary Table 3.


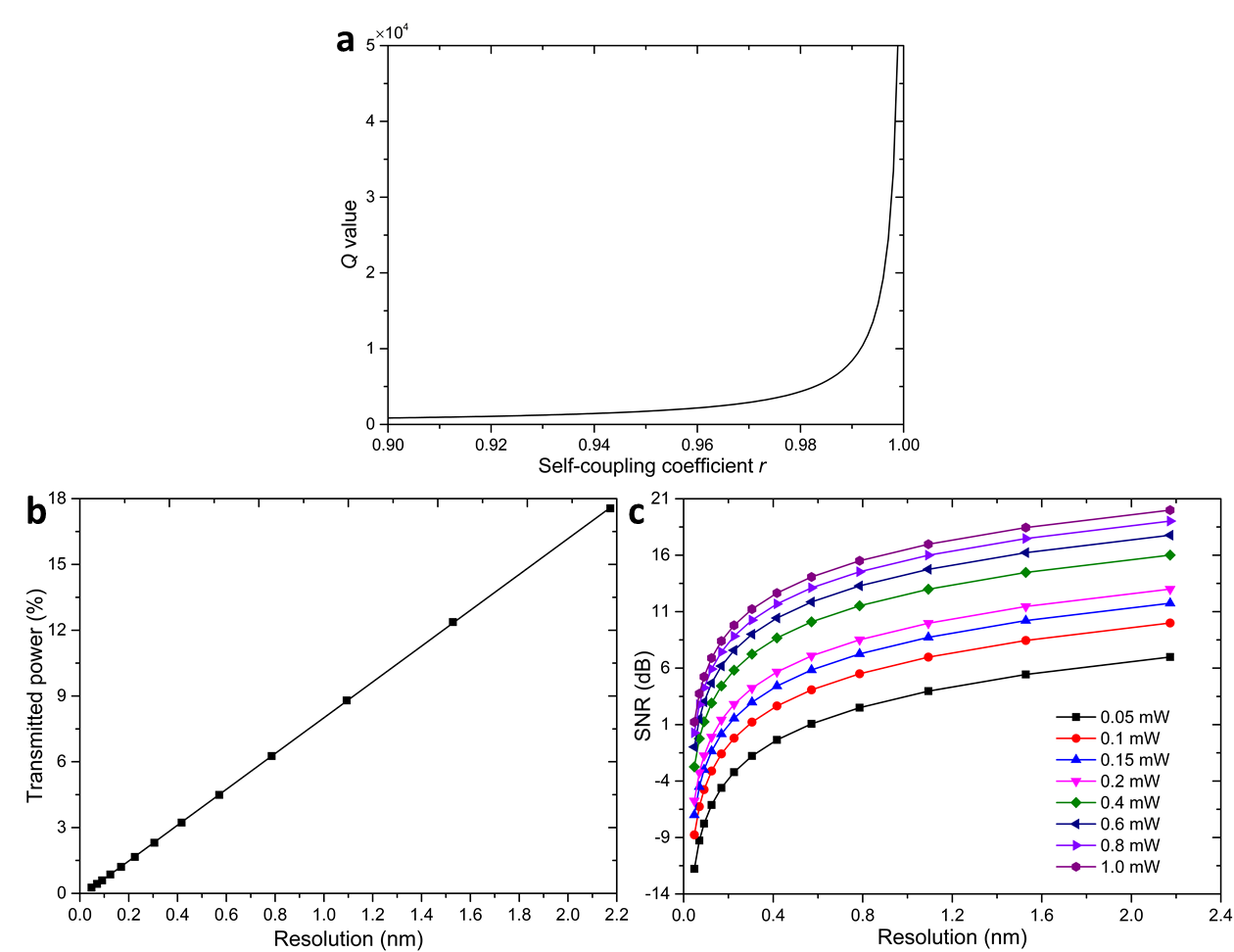


**Supplementary Figure 16.**  Ring coupler Analysis. **a** The Q value is proportional to r. **b** Simulated relation between transmitted power carried by the filtered sparse spectrum (m = 3) and resolution. **c** Simulated relation between SNR and resolution at different input power values.

| Parameter | *n*_g_ | *λ*_r_ | *L* | *a* |
| --- | --- | --- | --- | --- |
| value | 4.25 | 1528.256 nm | 20.734 μm | 0.9986 |

**Supplementary Table 3.** Parameters values for simulations in Supplementary Fig. 16.

**Supplementary Note 6 Experimental setup**

**
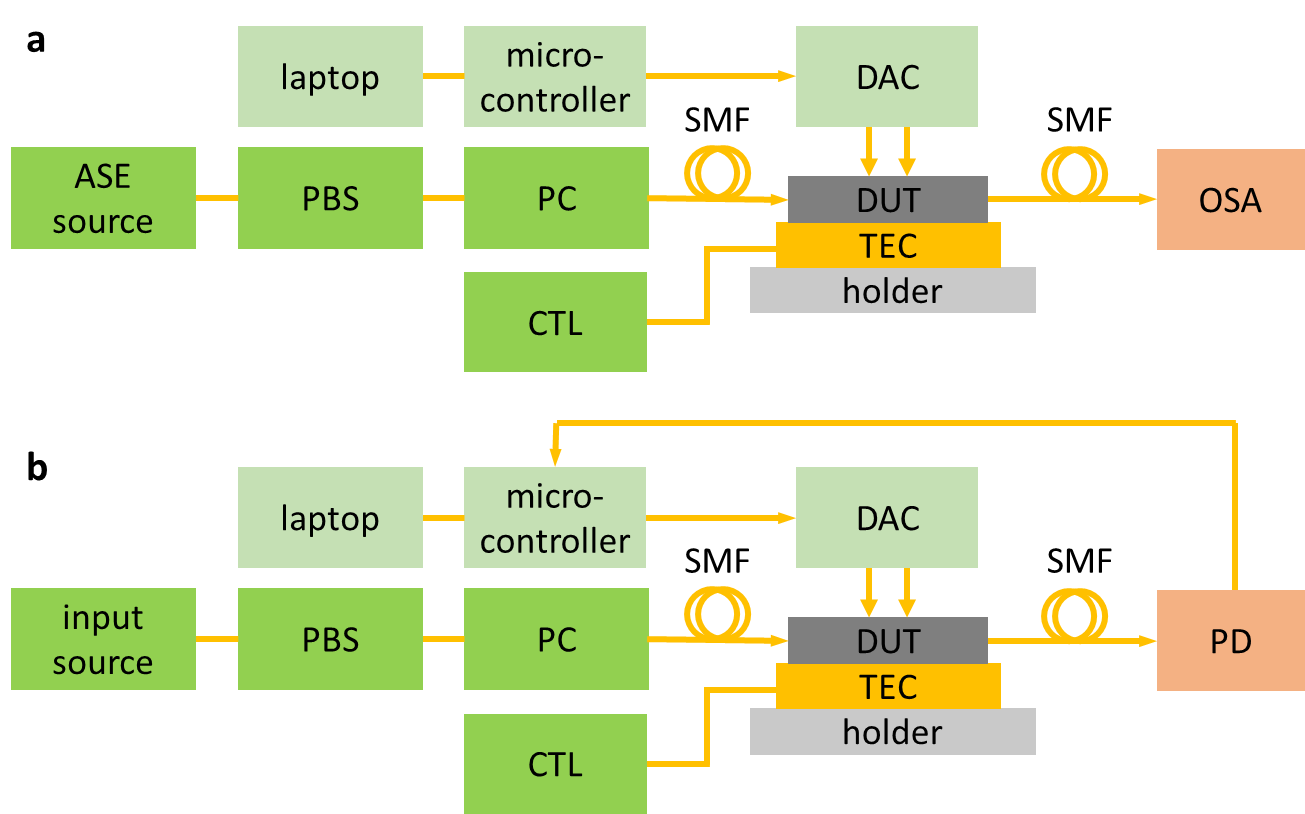
**

**Supplementary Figure 17.** Experimental setup. **a** MRR characterization. **b** Single and double wavelength characterization and broadband spectrum recovery.

**Supplementary References**

1. Frey, B. J., Leviton, D. B., & Madison TJ. Temperature-dependent refractive index of silicon and germanium. *Optomechanical Technologies for Astronomy,* 6273 II, 62732 J (2006).

2. Leviton, D. B., & Frey, B. J. Temperature-dependent absolute refractive index measurements of synthetic fused silica. *Optomechanical Technologies for Astronomy,* 6273 II, 62732 K (2006).

3. Souza, M. C., Grieco, A., Frateschi, N. C., & Fainman, Y. Fourier transform spectrometer on silicon with thermo-optic non-linearity and dispersion correction. *Nature communications* **9**, 665 (2018).

4. Della Corte, F. G,, Esposito Montefusco, M., Moretti, L., Rendina, I., Cocorullo, G. Temperature dependence analysis of the thermo-optic effect in silicon by single and double oscillator models. *J. Appl. Phys.* **88**, 7115-7119 (2000).

5. Okada, Y., & Tokumaru, Y. Precise determination of lattice parameter and thermal expansion coefficient of silicon between 300 and 1500 K. *J. Appl. Phys.* **56**, 314-320 (1984).

6. Rabus, D. G. *Integrated Ring Resonators: The Compendium*, (Springer, Berlin, 2007).

7. Fang, Q., et al. High efficiency ring-resonator filter with NiSi heater. *Ieee Photonic Tech. L.* **24**, 350-352 (2012).

8. Dong, P., et al. Thermally tunable silicon racetrack resonators with ultralow tuning power. *Opt. Express* **18**, 20298-20304 (2010).

9. Bogaerts, W., et al. Silicon microring resonators. *Laser Photonics Rev.* **6**, 47-73 (2012).
